# Rapid Genomic Characterization of High-Risk, Antibiotic Resistant Pathogens Using Long-Read Sequencing to Identify Nosocomial

**DOI:** 10.1017/ash.2024.255

**Published:** 2024-09-16

**Authors:** Chin-Ting Wu, Amy Spallone, Sherry Cantu, Todd Treangen, William Shropshire, Micah Bhatti, Israel Glover, Xiaojun Liu, Samuel Shelburne, Awdhesh Kalia

**Affiliations:** School of Health Professions, The University of Texas MD Anderson Cancer Center, Houston, TX; The University of Texas MD Anderson Cancer Center; MD Anderson Cancer Center; Department of Computer Science, Rice University

## Abstract

**Background:** Current epidemiological methods have limitations in identifying transmission of bacteria causing healthcare-associated infections (HAIs). Recent whole genome sequencing (WGS) studies found that genetically related strains can cause HAIs without meeting standard epidemiologic definitions, but these results could not provide data in a timely fashion needed for intervention. Given recent advances in Oxford Nanopore Technologies (ONT) sequencing, we sought to establish a validated ONT pipeline capable of providing accurate WGS-based comparisons of clinical pathogens within a short time frame that would allow for infection control interventions. **Method:** Using electronic medical record data, we identified potential healthcare acquisition of methicillin-resistant Staphylococcus aureus (MRSA), vancomycin-resistant enterococci (VRE), and carbapenem-resistant gram-negative rods. Bacterial genomic DNA was directly extracted from clinical microbiology lab plates. Sequencing was conducted with the ONT MinION sequencer and R10.4.1 flow cell. MINTyper for single nucleotide polymorphism (SNP) calling and Ridom SeqSphere+ for core genome MLST were used to determine genetic relatedness. The main outcome was time from pathogen identification to completed genetic analysis. **Result:** The weekly workflow, from genomic DNA extraction to complete data analysis, averaged 2.6 days with a standard deviation of 1.3 days. (range: 1 to 6 days). Starting in August 2023, we have sequenced a total of 177 bacterial isolates from 156 unique patients. Isolates came from blood (38%), tissue/wound/body fluid (24%), urinary tract (20%), respiratory tract (16%), and rectal swab (2%). To date, six genetically related clusters have been identified. Three clusters involved ST117 vancomycin-resistant Enterococcus faecium (VREfm), comprising a total of 13 unique patients distributed as 2, 3, and 8 patients in each group, with pairwise SNP differences of 20, 11, and 14. Patients within the same clusters showed epidemiological links through overlapping admissions and temporally shared ICU stays. Additionally, another cluster consisted of five genetically related ST633 Pseudomonas aeruginosa isolates, with a pairwise SNP difference of 57.5. Each patient in this cluster had potential epidemiological links through overlapping admission times, despite the absence of identified shared spaces. The last two clusters involved Klebsiella pneumoniae and Escherichia coli (two cases each), with pairwise SNP differences of 18 and 9, respectively. In both cases, each patient showed potential epidemiological links through overlapping admission times. **Conclusion:** Our stand-alone ONT pipeline was able to rapidly and accurately detect genetically related AMR pathogens, aligning closely with epidemiological data. Our approach has the potential to assist in the efficient detection and deployment of preventative measures against healthcare-associated infection transmission.

